# 25th Anniversary of Molecules—Recent Advances in Green Chemistry

**DOI:** 10.3390/molecules26123768

**Published:** 2021-06-21

**Authors:** Gonzalo de Gonzalo, Mara G. Freire

**Affiliations:** 1 Departamento de Química Orgánica, Universidad de Sevilla, 41014 Sevilla, Spain; 2 Chemistry Department, CICECO—Aveiro Institute of Materials, University of Aveiro, 3810-193 Aveiro, Portugal

Green Chemistry has been defined by the EPA as the design of chemical products and processes that reduce or eliminate the use or generation of hazardous substances. This approach reduces the negative impact of chemistry on human health and the environment by focusing on all of the life cycle of given a chemical product, including its design, synthesis, application and disposal. In the last few years, this sustainable approach has gained an overwhelming interest, as can be observed by the significant number of publications devoted to the development of greener chemicals and greener processes.

Since its first application in organic synthesis, biocatalysis, that is the use of a biological catalysts for the preparation of valuable compounds, has been demonstrated to be one of the most sustainable approaches in asymmetric catalysis. Furthermore, mild reaction conditions and greener reagents, including the use of aqueous media, have led to relevant advantages in a wide diversity of reactions. These two different approaches are shown in the present Special Issue. One work is focused on the application of biocatalysis in the production of bulk compounds and other related products [1], whereas the other analyzes the recent developments in sustainable reaction media when developing biocatalytic redox processes, using neat conditions, green solvents and deep eutectic solvents (DES) [2]. These last solvents, which are easy to prepare and if properly selected may show a high degradability and low toxicity, have been widely employed in chemistry with different purposes, as shown in extraction strategies for the determination of insecticide residues in water, soil and egg yolk samples [3], or for the recovery and stabilization of anthocyanins and phenolic oxidants of Roselle [4]. Supercritical solvents have also been employed in green chemistry, being one of the most relevant classes of green solvents, as shown with the modification of commercial cellulose acetate microfiltration membranes by supercritical solvent impregnation in the presence of thymol, with the aim of generating membranes with antibacterial properties [5]. Polymers and their application in green chemistry processes are also presented in this present Special Issue. Recent developments in biopolymers and conducting-based polymers as sensors for the determination of pollutants are here described [6] as novel methods for the chemical recycling of end-use poly (ethylene terephthalate) (PET) in batch, microwave and electrochemical reactors [7], and for the preparation of organic polymers via continuous or discontinuous expansion processes in supercritical CO_2_ [8]. Aspects related with the determination of pollutants as persistent organic pollutants or polycyclic aromatic hydrocarbons in different samples are also covered [9,10]. Finally, this collection provides information on the stability to enzymatic degradation of the tricyclic acyclovir derivative and its esters (acetyl, isobutyryl, pivaloyl, nicotinic, ethoxycarbonyl) compared with the stability of analogue acyclovir esters [11], as well as a mechanistic study on homogenous charge compression ignition engines [12]. The twelve manuscripts published in the current Special Issue cover almost all aspects of Green Chemistry. Although there is still a long path ahead to fulfill all requirements of Green Chemistry in our society, the results here presented disclose the recent potential and efforts being accomplished towards this goal.
